# Mechanism of lily bulb and Rehmannia decoction in the treatment of lipopolysaccharide-induced depression-like rats based on metabolomics study and network pharmacology

**DOI:** 10.1080/13880209.2022.2121843

**Published:** 2022-10-07

**Authors:** Xiansu Chi, Xiaoyan Xue, Jin Pan, Jiang Wu, Huishan Shi, Yong Wang, Yanting Lu, Zhe Zhang, Ke Ma

**Affiliations:** a Xiyuan Hospital, China Academy of Chinese Medical Sciences, Beijing, PR China; b Shandong Co-Innovation Center of Classic TCM Formula, Shandong University of Traditional Chinese Medicine, Jinan, PR China

**Keywords:** Traditional Chinese medicine formula, depressive disorder, medial prefrontal cortex, metabolic components

## Abstract

**Context:**

Lily bulb and Rehmannia decoction (LBRD), consisting of *Lilium henryi* Baker (Liliaceae) and *Rehmannia glutinosa* (Gaertn) DC (Plantaginaceae), is a specialized traditional Chinese medicine formula for treating depression. However, the underlying mechanisms, especially the relationship between LBRD efficacy and metabolomics, remains unclear.

**Objective:**

This study was aimed to investigate the metabolic mechanism of LBRD in treating depression.

**Materials and methods:**

Network pharmacology was conducted using SwissTargetPrediction, DisGeNET, DrugBank, Metascape, etc., to construct component-target-pathway networks. The depression-like model was induced by intraperitoneal injection with lipopolysaccharide (LPS) (0.3 mg/kg) for 14 consecutive days. After the administration of LBRD (90 g/kg) and fluoxetine (2 mg/kg) for 14 days, we assessed behaviour and the levels of neurotransmitter, inflammatory cytokine and circulating stress hormone. Prefrontal metabolites of rats were detected by using liquid chromatography–mass spectrometry metabolomics method.

**Results:**

The results of network pharmacology showed that LBRD mainly acted on neurotransmitter and second messenger pathways. Compared to the model group, LBRD significantly ameliorated depressive phenotypes and increased the level of 5-HT (13.4%) and GABA (24.8%), as well as decreased IL-1β (30.7%), IL-6 (32.8%) and TNF-α (26.6%). Followed by LBRD treatment, the main metabolites in prefrontal tissue were contributed to retrograde endocannabinoid signalling, glycerophospholipid metabolism, glycosylphosphatidylinositol-anchor biosynthesis, autophagy signal pathway, etc.

**Discussion and conclusions:**

LBRD were effective at increasing neurotransmitter, attenuating proinflammatory cytokine and regulating glycerophospholipid metabolism and glutamatergic synapse, thereby ameliorating depressive phenotypes. This research will offer reference for elucidating the metabolomic mechanism underlying novel antidepressant agents contained LBRD formula.

## Introduction

Depression is a chronic emotional disorder accompanied by symptoms ranging from, but not limited to, restlessness, fluctuated appetite, unclear consciousness, frequent silence, difficulty in concentration, and lack of interest (Liang et al. 2018; Liu et al. 2020). Without doubt, depression is a multifactorial disorder that adversely affects social interpersonal communication and general health (Wu et al. 2016), with complex, multifactorial aetiology and complicated clinical manifestations, therefore, it has become a hot public issue. Research has demonstrated that its pathogenesis is related to disorders of monoamine transmitters, changes in the function of the hypothalamic-pituitary-adrenal (HPA) axis, imbalance of the glutaminergic neurotransmitter system, changes in the structure and function of the brain, genetic and epigenetic changes in the inflammatory response, and environmental factors (Palucha and Pilc 2005; Malhi and Mann 2018). It is reported that prolonged exposure to inflammatory mediators impairs the regulation of neuroendocrine stress, affects the availability of monoamine neurotransmitters, and may therefore contribute directly to depressive symptoms (Anisman et al. 2002; Maes et al. 2009; Miller et al. 2009). More than 300 million people of all ages suffer from depressive disorders worldwide, and up to 40% do not adequately respond to antidepressant medications (Holtzheimer and Mayberg 2011). Attention should be paid to this large number of patients and the high incidence of suicide, hence, multi-target antidepressants with high efficiency and low toxicity should be developed (Hughes et al. 2017; Read et al. 2017; Dodd et al. 2018).

Based on the characteristics of multiple components, targets and overall regulation, traditional Chinese medicine (TCM) has unique advantages and great prospects in the prevention and treatment of diseases. Lily bulb and Rehmannia decoction (LBRD), a TCM classic formula, first recorded in the *Synopsis of the Golden Chamber: Lily Disease, Huhuo and Yin Yang Toxin: Pulses, Syndromes and Treatment*, has long been used as a specialized formula for treating lily disease, an emotional disease in TCM, with the mechanism of yin deficiency and internal heat. LBRD, consisting of *Lilium henryi* Baker (Liliaceae) and raw root juice from *Rehmannia glutinosa* (Gaertn) DC (Plantaginaceae), decocted with spring water, functions in nourishing yin, clearing heat, and moistening the lung and heart to improve and relieve depression-like symptoms (Li et al. 2012; Zhang et al. 2020). Although clinical and experimental studies have demonstrated that LBRD is a well-known TCM formula for treating depression, there are few reports on its efficacy in treating depression from the perspective of metabolism.

In this study, we revealed the mechanism of LBRD in the treatment of lipopolysaccharide (LPS)-induced depression-like rats from the perspectives of metabolism and network pharmacology. First, network pharmacology analysis was used to demonstrate the multiple compounds and targets of LBRD and to investigate the potential signalling pathways. Second, changes in the behavioural tests, neurotransmitter levels and inflammatory cytokine levels were assessed. Finally, through a metabolic study, differentially expressed metabolites and metabolic pathways were identified.

Through these comparisons and analyses, we expect to decipher the main metabolites, metabolic pathways and pathogenesis involved in the antidepressant effects of LBRD. This research will provide a reference for the clinical application and modernization of TCM classic formulas and provide an experimental basis for the application of LBRD in patients with depression.

## Materials and methods

### Network pharmacology analysis

To reveal the underlying biological mechanism by which LBRD regulates the depression, network pharmacology was applied to analyse the targets and pathways in which LBRD interfered with the regulation of depression. A chemistry database was searched to collect the information on compounds in LBRD. The active compounds were predicted using SwissTargetPrediction, and compounds with probability ≥0.1 were selected. The corresponding targets of the compounds from LBRD were predicted using DisGeNET, DrugBank, OMIM and TTD to identify whether these targets were related to depression. The targets corresponding to the effective ingredients of LBRD and those associated with depression were compared to determine the common targets. Metascape was used to perform pathway enrichment and Cytoscape 3.7.0 software was used to visualize the network between LBRD and depression.

### Animals and drug administration

The experimental protocol was devised in accordance with the NIH Guidelines for Care and Use of Laboratory Animals and approved by the Animal Care and Animal Ethics Committee of Shandong University of Traditional Chinese Medicine (SDUTCM201805311223). Healthy specific pathogen free male Sprague-Dawley rats (aged 5 weeks, body weight 180–200 g) were obtained from Beijing Vital River Laboratory Animal Technology Co., Ltd. (Beijing, China). All the rats were housed in cages and maintained at a controlled temperature (22 ± 2 °C) with humidity of 60 ± 5%, under a 12 h light–dark cycle with free access to food and water. After a three-day acclimatization period, all rats were randomly divided into the following groups (*n* = 12): (1) control group, (2) model group (LPS), (3) LPS + LBRD group and (4) LPS + fluoxetine (Flu) group.

**Figure 1.**
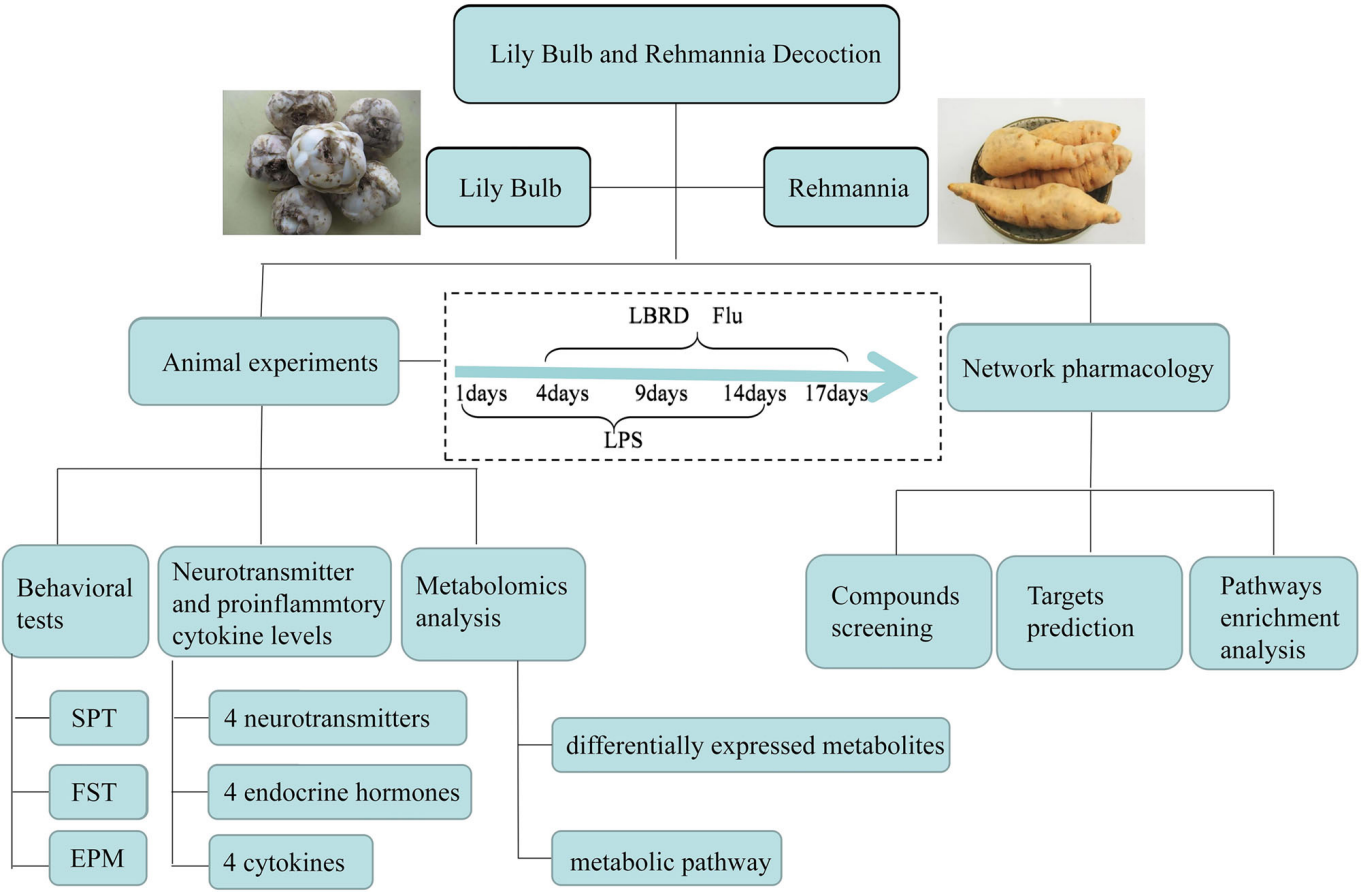
The main experimental timeline and test indicators of combined mechanisms of LBRD on depression. After a three days acclimatization period, rats were divided into four groups. LPS was injected for 14 days to establish the depression model. Three days after the initiation of the injection of LPS, the intervention of medicine was conducted for 14 days. Animal experiments and network pharmacology were used to classify the mechanism.

LPS was dissolved in sterile, endotoxin-free isotonic saline and administered by intraperitoneal injection at a dose of 0.3 mg/kg to the rats in the model, LPS + LBRD and LPS + Flu groups for 14 days. Rats in the control group were injected with an equivalent volume of saline water without LPS for 14 days. After intervention with LPS for 3 days, the rats in the LPS + LBRD and LPS + Flu groups were administrated with the LBRD (90.0 g/kg) and Flu (2 mg/kg), respectively, at a volume of 5 mL/kg by gavage 2 h after the injection of LPS for 14 consecutive days (2 weeks). At the same time, rats in the control and model groups were administered an equivalent volume of saline water by gavage 2 h after the injection of LPS. A detailed experimental timeline is shown in Figure 1.

### Preparation of LBRD

The genuine production area of the lily bulb, raw Rehmannia root and the conversion of the original doses to the modern volume and the preparation process of LBRD were reported in our previous study (Chi et al. 2019). In this study, we used the lily bulb from Shennongjia (Hubei Province, China) and fresh Rehmannia root from Jiaozuo (Henan Province, China). The above two herbs were authenticated by S. Ma, the Professor of Shandong University of Traditional Chinese Medicine. The specimens of lily bulb (no. SDUTCM, 20201009-01) and raw Rehmannia root (no. SDUTCM, 20201015-03) were collected in December 2020 and stored at Shandong University of Traditional Chinese Medicine.

LBRD was prepared as follows: 490 g lily bulb was decocted in 400 mL spring water to obtain a 200 mL decoction, and 400 g fresh Rehmannia root was sliced and squeezed to obtain 200 mL juice. Both were mixed, and the mixture was further decocted with mild fire to obtain a 300 mL decoction for oral administration.

### Behavioural tests

The sucrose preference test (SPT) and forced swimming test (FST) were used to assess anhedonia behaviour and behavioural despair, respectively, and the elevated plus maze (EPM) test was used to evaluate social interaction behaviour. Depressive-like behaviours were assessed 24 h after the intervention to determine whether LPS-induced depression-like rats showed depression-like behaviours and the intervention effects of LBRD on depressive-like behaviours. All behavioural tests were performed between 9:00 am and 17:00 pm under dim light and low noise conditions. Behaviours were monitored by well-trained observers blinded to the treatment method. The animals were sacrificed after the behavioural tests were completed.

### Measurement of neurotransmitter and proinflammatory cytokine levels

As soon as the behavioural tests were completed, the rats were anaesthetized with 2% pentobarbital sodium (40 mg/kg) by intraperitoneally injection. Blood samples were taken from the abdominal aorta, collected in a vacuum blood collection tube (containing separating gel and coagulant), centrifuged at 5000 rpm for 15 min, transferred into 1.5 mL Eppendorf tubes, and stored at −80 °C. Enzyme-linked immunosorbent assay (ELISA) was used to classify the levels of neurotransmitters and proinflammatory cytokines.

### Methods of liquid chromatography–mass spectrometry metabolomics analysis

After the behavioural tests, all rats were sacrificed simultaneously and the medial prefrontal cortex (mPFC) were separated quickly and frozen at −80 °C. Brain tissue samples were diluted with an internal standard solution, and a 1 mL methanol–acetonitrile mixture (4:1, v/v). The samples were homogenized using a tissue grinder and centrifuged at 14,000 rpm at 4 °C for 15 min, and the supernatant was collected and lyophilized. After reconstitution in 100 µL of methanol–acetonitrile mixture (4:1) and centrifugation at 14,000 rpm for 15 min at 4 °C, the supernatant was filtered through a 0.22 μm microfilter for liquid chromatography–mass spectrometry metabolomics (LC–MS) analysis. From each sample, 10 μL of supernatant was mixed and used as the quality control sample.

An ACQUITY UHPLC system (Waters Corporation, Milford, MA) with a column (1.7 μm, 2.1 mm × 100 mm) was used for liquid chromatography separation. The AB SCIEX Triple TOF 5600 System (AB SCIEX, Framingham, MA) was used to analyse the metabolic profiles. The raw data were analysed using Progenesis QI software. Water (containing 0.1% formic acid, v/v) (A) and acetonitrile (containing 0.1% formic acid, v/v) (B) made up the binary gradient elution system and separation was achieved using the following gradient: 0 min, 5% B; 2 min, 20% B; 4 min, 60% B; 11 min, 100% B; 13 min, 100% B; 13.5 min, 5% B and 14.5 min, 5% B. The flow rate was controlled at 0.4 mL/min and the column temperature was set at 40 °C. The mass spectrometer operating parameters were conducted as follows: the ion source temperature was 550 °C (+) and 550 °C (–), the ion spray voltage was 5500 V (+) and 4500 V (–), the curtain gas of 35 PSI was 100 V (+) and −100 V (–), the collision energy was 10 eV (+) and −10 eV (–) and the interface heater temperature was 550 °C (+) and 600 °C (–).

### Data analysis

Data from the behavioural tests and the levels of neurotransmitter, inflammatory cytokine and circulating stress hormone were analysed using the SPSS 26 statistical package (SPSS Inc., Chicago, IL), and the results were expressed as means ± standard deviation (SD). The means between two groups were compared by Student’s *t*-test, and means between three or more groups were compared by one-way analysis of variance (ANOVA) followed by Dunnett’s test. Differences were considered significant at a level of *p*< 0.05.

The transformed data from LC–MS metabolomics analysis were imported into the SIMCA software (version 14.0; Umetrics, Umeå, Sweden). Orthogonal partial least-squares discriminant analysis (OPLS-DA) was conducted to visualize the differences in the levels of metabolite levels between the groups. Variable importance in the projection (VIP) ranks the overall contribution of each variable to the OPLS-DA model, and those variables with a VIP > 1 are considered relevant for group discrimination. Metabolites with fold change between two groups ≥ 1.5 were selected as the metabolites with significant differences. Differential metabolites (VIP > 1.0, *p* < 0.05) detected by LC–MS were identified by searching the LIPIDMAPS, METLIN database and Human Metabolome Database (Wishart et al. 2013). These databases were linked to KEGG, PubChem, LIPIDMAPS and ChEBI for further analysis of metabolic pathways (Durani et al. 2017).

## Results

### Network pharmacology analysis for the antidepressant effect of LBRD

After the analysis of the compounds of LBRD, 33 active compounds were selected, among them, there were 4 active compounds from lily bulb, and 29 active compounds from Rehmannia. Four online target prediction servers were used to predict the targets of active compounds. Finally, 191 targets were identified, including 56 targets from lily bulb and 175 targets from Rehmannia (among them, 40 targets were the common targets) were determined.

**Figure 2.**
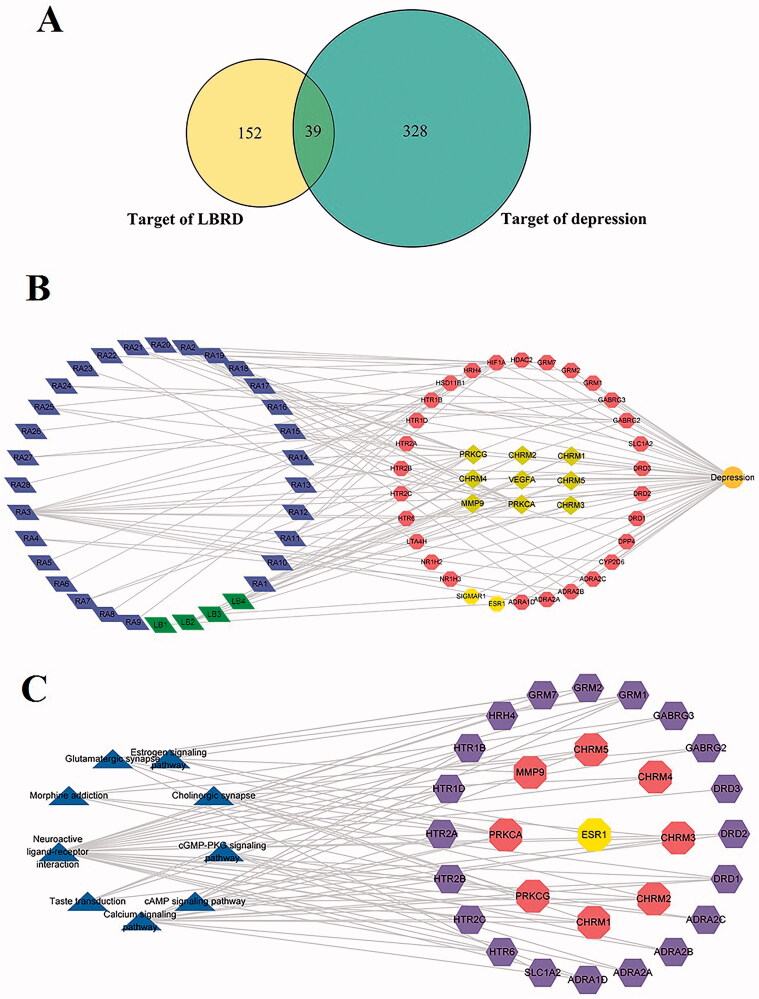
LBRD attenuates depression via the multiple compounds, multiple targets and the several signalling pathways. (A) The common 39 targets between the 191 targets of LBRD and the 367 targets of the depression. (B) The active components and target genes of LBRD for treating depression. The green diamond represents the active ingredient of lily bulb, the blue diamond represents the active ingredient of Rehmannia, the yellow hexagon represents the effective antidepressant target of lily bulb, and the red hexagon represents the effective antidepressant target of Rehmannia. The yellow diamond represents the common antidepressant target of lily bulb and Rehmannia. (C) Target-pathway networks of LBRD. The triangles represent the enriched pathways, the red hexagons represent the common antidepressant target of lily bulb and Rehmannia, the purple hexagons represent the effective antidepressant target of Rehmannia, and the yellow hexagons represent the effective antidepressant target of lily bulb.

To further explore the mechanisms of LBRD for anti-depression, 367 targets of depression were predicted using four disease databases. The 191 targets from LBRD and 367 targets from the depression were compared to obtain 39 common targets (Figure 2(A)). To further explore the anti-depression mechanisms of LBRD, a component-target-disease-gene network (consisting of 72 nodes and 127 edges) was constructed (Figure 2(B)). The results showed that LBRD involved 32 effective components and 39 target genes for treating depression.

To further clarify the pathways by which LBRD treats depression, in the KEGG database, pathways involving targets with *p* < 0.01 were identified (Figure 2(C)). After enrichment analysis, the top nine pathways were selected, resulting in a target-pathway (T-P) network. The T-P network contained 38 nodes (nine pathways, 29 targets). Among them, 20 targets were shared by the LBRD, with eight targets unique to Rehmannia and one target unique to lily bulb. The associated signalling pathways, including the neuroactive ligand–receptor interaction, calcium signalling pathway, cholinergic synapse, glutamatergic synapse, morphine addiction, taste transduction, oestrogen signalling pathway, cyclic adenosine monophosphate (cAMP) signalling pathway and cyclic guanosine monophosphate (cGMP)-protein kinase G (PKG) signalling pathway, exhibited a relatively high number of target connections. These results proved that the antidepressant effect of LBRD was in a combined form.

### The alleviated effect of LBRD on depression-like behaviours in LPS-induced depression-like rats

**Figure 3.**
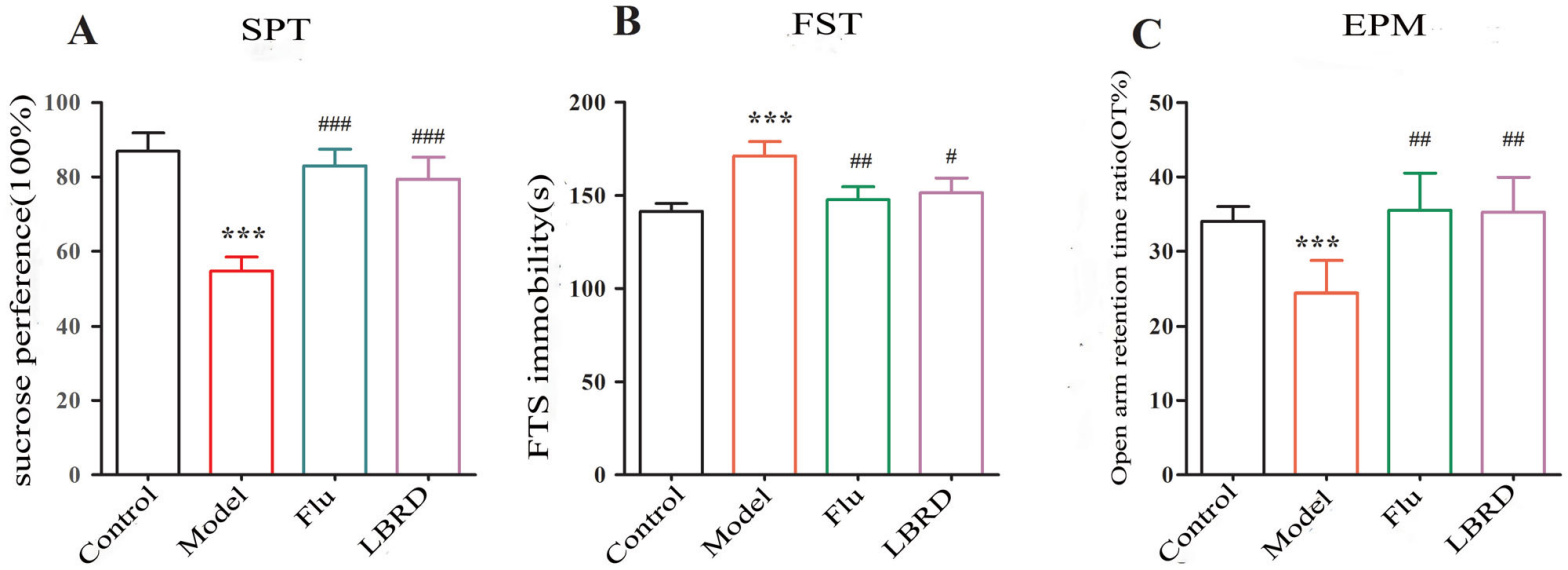
LBRD standard decoction attenuates the depressive and anxiety symptoms in LPS-induced depression-like rats. (A) Representation of the percent of sucrose consumption/total water intake in the SPT. (B) Representation of the immobility time of rats in FST. (C) Open arm retention time ratio in the EPM test (*n* = 8–10 per group) and differences between two groups were compared by Student’s *t*-test. ***p< 0.001, vs. control group; ^#^*p*< 0.05, ^##^*p*< 0.01, ^###^*p*< 0.001, vs. the model group. Control: control group, Model: model group (LPS), Flu: LPS + Flu group and LBRD: LPS + LBRD group.

The sucrose preference, immobility time in the FST, and open arm retention time ratio in the EPM test were used to evaluate the depressive manifestation of LPS-induced depression-like rats and assess the intervention effects of LBRD on depressive-like behaviours. With respect to changes in the sucrose preference, 2 weeks after LPS injection, the sucrose preference of the model group was significantly lower than that of the control group (*p* < 0.01). After 2 weeks of treatment, the LPS + LBRD and LPS + Flu groups showed a dramatic increase in sucrose intake compared to the model group (*p* < 0.01) (Figure 3(A)). As shown in Figure 3(B), the immobility time of rats in the FST was increased after LPS-inducement (*p* < 0.01), indicating that the intervention with LPS could induce depressive symptoms. After 2 weeks of treatment, the intervention of LBRD and Flu significantly attenuated the increase in immobility time observed in LPS-induced depression-like rats (*p* < 0.05 and *p* < 0.01, respectively). In terms of the open arm retention time ratio, the induction of LPS significantly decreased the ratio of the open arm retention time, and the intervention of LBRD and Flu reversed this decrease (*p* < 0.01 and *p* < 0.01, respectively) (Figure 3(C)). We tentatively concluded from behavioural performance that LPS could induce depressive symptoms, and LBRD could relieve depressive symptoms.

### The effect of LBRD on neurotransmitter and proinflammatory cytokine levels in LPS-induced depression-like rats

**Figure 4.**
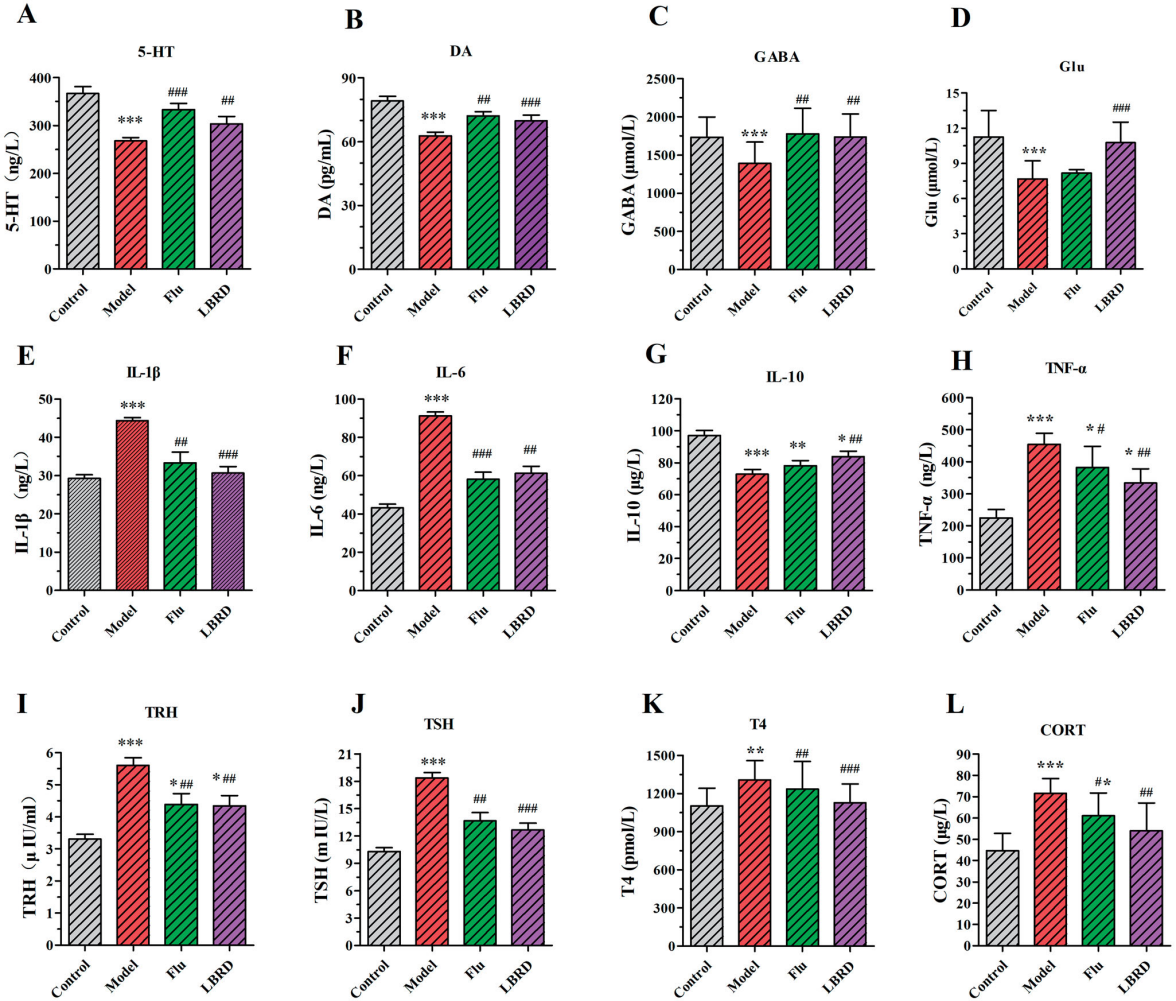
LBRD ameliorates neurotransmitter and inflammatory cytokine levels in the serum of rats induced by LPS. LBRD standard decoction or fluoxetine treatment significantly increased the level of 5-HT (A), DA (B), GABA (C), IL-10 (G), and decreased Glu (D), IL-1β (E), IL-6 (F), TNF-α (H), TRH (I), TSH (J), T4 (K) and CORT (L). Data are expressed as mean ± SD (*n* = 8–10 per/group) and differences between two groups were compared by Student’s *t*-test. *p< 0.01, **p< 0.01, ***p< 0.001, vs. control group; ^#^*p*< 0.05, ^##^*p*< 0.01, ^###^*p*< 0.001, vs. model group. Control: control group, Model: model group (LPS), Flu: LPS + Flu group and LBRD: LPS + LBRD group.

The effect of the LBRD standard decoction on the level of neurotransmitter and inflammatory cytokines was investigated by ELISA. After 4 consecutive weeks of model construction, serum samples from each group were collected, and 12 biochemical factors were detected by ELISA, including four neurotransmitters, four endocrine hormones and four cytokines (Figure 4). The levels of 5-hydroxytryptamine (5-HT), dopamine (DA) and γ-aminobutyric acid (GABA) were notably decreased in the model group compared with those in the control group (Figure 4(A–C), model group vs. control group, 5-HT: 268.00 ± 7.48 vs. 367.00 ± 14.10, *p* < 0.01; DA: 62.74 ± 1.776 vs. 79.21 ± 2.099, *p* < 0.01; GABA: 1389.00 ± 280.10 vs. 1729.00 ± 268.50, *p* < 0.01). In contrast, LBRD could effectively restore the attenuation (Figure 4(A–C), LPS + LBRD group vs. model group, 5-HT: 304.00 ± 15.40 vs. 268.00 ± 7.48, *p* < 0.01; DA: 69.80 ± 2.702 vs. 62.74 ± 1.776, *p* < 0.001; GABA: 1734.00 ± 304.80 vs. 1389.00 ± 280.10, *p* < 0.01). Regarding the level of glutamate (Glu), LPS-induced depression-like rats demonstrated a significant decrease in Glu levels, and the intervention of LBRD significantly attenuated the decreased level of Glu (Figure 4(D), LPS + LBRD group vs. model group, Glu: 10.75 ± 1.769 vs. 7.671 ± 1.532, *p* < 0.001). Compared to the control group, the serum levels of interleukin-1β (IL-1β), interleukin-6 (IL-6) and tumour necrosis factor-α (TNF-α) in the model group were significantly increased (Figure 4(E,F,H), model group vs. control group, IL-1β: 44.3 ± 0.842 vs. 29.3 ± 0.934, *p* < 0.001; IL-6: 91.27 ± 1.992 vs. 43.33 ± 1.895, *p* < 0.001; TNF-α: 454.5 ± 34.69 vs. 224.4 ± 26.80, *p* < 0.001), and the level of interleukin-10 (IL-10) was significantly decreased (Figure 4(G), model group vs. control group, IL-10: 72.83 ± 3.03 vs. 97.19 ± 3.052, *p* < 0.001). After the intervention with LBRD, alternations in the levels of IL-1β, IL-6, TNF-α and IL-10 were restored (Figure 4(E–H), LPS + LBRD group vs. model group, IL-1β: 30.7 ± 1.73 vs. 44.3 ± 0.842, *p* < 0.001; IL-6: 61.30 ± 3.612 vs. 91.27 ± 1.992, *p* < 0.01; TNF-α: 333.8 ± 43.84 vs. 454.5 ± 34.69, *p* < 0.01; IL-10: 83.97 ± 3.344 vs. 72.83 ± 3.03, *p* < 0.01).

After the administration of LPS, the levels of endocrine hormones including thyroid stimulating hormone releasing hormone (TRH), thyroid stimulating hormone (TSH), thyroxine (T4) and cortisol (CORT) were higher than those in the control group (Figure 4(I–L), model group vs. control group, TRH: 5.61 ± 0.236 vs. 3.31 ± 0.148, *p* < 0.001; TSH: 18.35 ± 0.6188 vs. 10.31 ± 0.4156, *p* < 0.001; CORT: 71.61 ± 6.883 vs. 44.63 ± 8.071, *p* < 0.001; T4: 1307.00 ± 154.0 vs. 1104.00 ± 139.6, *p* < 0.01). Compared with the model group, the levels of TRH, TSH, T4 and CORT were significantly decreased after the intervention with LBRD (Figure 4(I–L), LPS + LBRD group vs. model group, TRH: 4.34 ± 0.316 vs. 5.61 ± 0.236, *p* < 0.01; CORT: 53.98 ± 13.00 vs. 71.61 ± 6.883, *p* < 0.01; TSH: 12.67 ± 0.7207 vs. 18.35 ± 0.6188, *p* < 0.01; T4: 1128.00 ± 148.9 vs. 1307.00 ± 154.0, *p* < 0.001).

### Multivariate statistical analysis of the metabolic data

**Figure 5.**
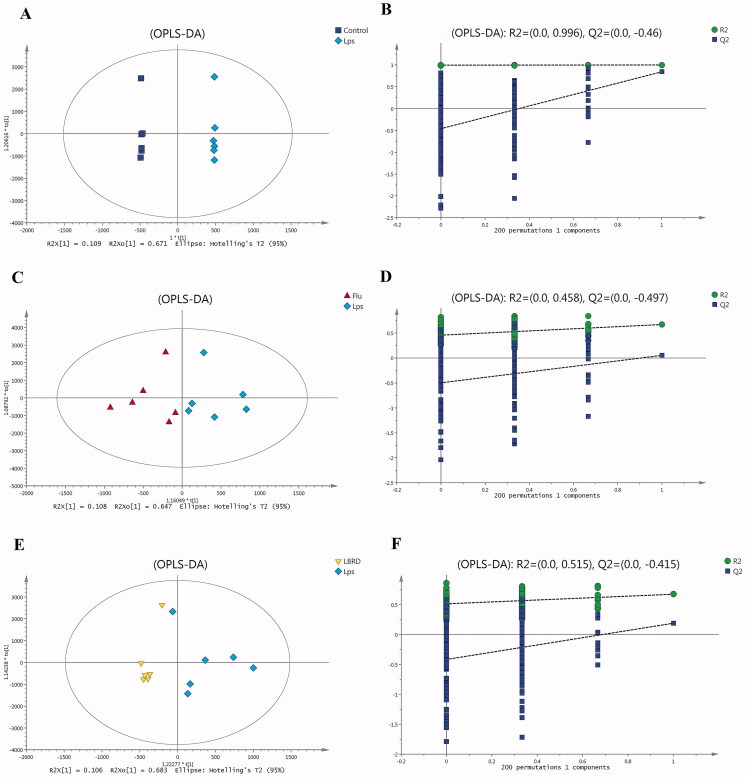
Metabolomic analysis of mPFC samples. (A) OPLS-DA score plot in the control and model groups (R_2_=0.981, Q_2_=0.844). (B) Permutation test in the control and model groups (R_2_=0.998, Q_2_=–0.46). (C) OPLS-DA score plot in the model group and LPS + Flu groups (R_2_=0.755, Q_2_=0.0524). (D) Permutation test in the model group and LPS + Flu group (R_2_=0.458, Q_2_=–0.497). (E) OPLS-DA score plot in the model group and LPS + LBRD group (R_2_=0.789, Q_2_=0.191). (F) Permutation test in the model group and LPS + LBRD group (R_2_=0.515, Q_2_=–0.415).

OPLS-DA is a statistical method of supervised discriminant analysis that can improve analytical ability and further improve the separation between the two groups. In the OPLS-DA score plot, as shown in Figure 5(A), a clear separation between the control and model groups was observed. This analysis showed that the LPS-treated rats were clearly discriminated from the healthy controls (R_2_X = 0.981, R_2_Y = 1, Q_2_=0.844), as these parameters approached 1.0, indicating a robust model with predictive reliability and explanatory capabilities. As shown in Figure 5(B), Q_2_= −0.46 suggests that the model was not over-fitting.

As shown in Figure 5(C), OPLS-DA loading plots showed a clear separation between the LPS + Flu and model groups (R_2_X = 0.755, R_2_Y = 0.671, Q_2_=0.0524). Q_2_ represents the cumulative predicted variation. It should be noted that the main automatic modelling parameters Q_2_ were smaller than 0.5; therefore, a permutation test should be performed to determine whether the model was over-fitting. As shown in Figure 5(D), Q_2_= −0.497 suggests that the model did not overfit. As shown in Figure 5(E), the LPS + LBRD group was separated from the model group with little overlap (R_2_X = 0.789, R_2_Y = 0.675, Q_2_=0.191). A permutation test was conducted to determine whether the model was over-fitting. As shown in Figure 5(F), Q_2_= −0.415 suggests that the model was not over-fitting.

Compared to the model group, the metabolites with significantly different changes after LPS induction are plotted in Figure 6(A). The results showed that the induction by LPS significantly affected the levels of 38 metabolites. Among them, 5 metabolites were down-regulated and other 33 metabolites were up-regulated. The names and other details of the corresponding metabolites are listed in Table 1. After LBRD intervention, seven metabolites were identified by comparing the differences between the model and LPS + LBRD groups (Figure 6(B)). The intervention of LBRD increased five metabolites and decreased two metabolites which are shown in Table 2. In addition, among these metabolites, 34 metabolites were classified into lipids and lipid-like molecules, consistent with previous preclinical and clinical experiments, suggesting that lipids are associated with neuronal function, and the composition of brain lipids may affect perception and emotional behaviour, resulting in depression and anxiety disorders (Adibhatla and Hatcher 2008; Yadav and Tiwari 2014; Kornhuber et al. 2015). Depression is characterized by disturbances in the central lipid metabolism (Zheng et al. 2012; Liu et al. 2015; Jia et al. 2016). Combined with changes in metabolites, our result suggested that the occurrence and treatment of depression may be associated with the glycerophospholipid metabolism.

Figure 6.The alerted metabolites and metabolic pathway in the comparison of different group. LBRD may play an antidepressant role through biological functions of the nervous system signalling/neurotransmitters, lipid metabolism, autophagy and virus infection. (A) Heat map of the differential metabolites in the mPFC (control group vs. model group) (*n* = 6 per/group). (B) Heat map of the differential metabolites in the mPFC (model group vs. LPS + LBRD group) (*n* = 6 per/group). (C) Summary of pathway analysis with metabolic analysis between control group and model group. (D) Summary of pathway analysis with metabolic analysis between model group and LPS + LBRD group.

**Figure:**
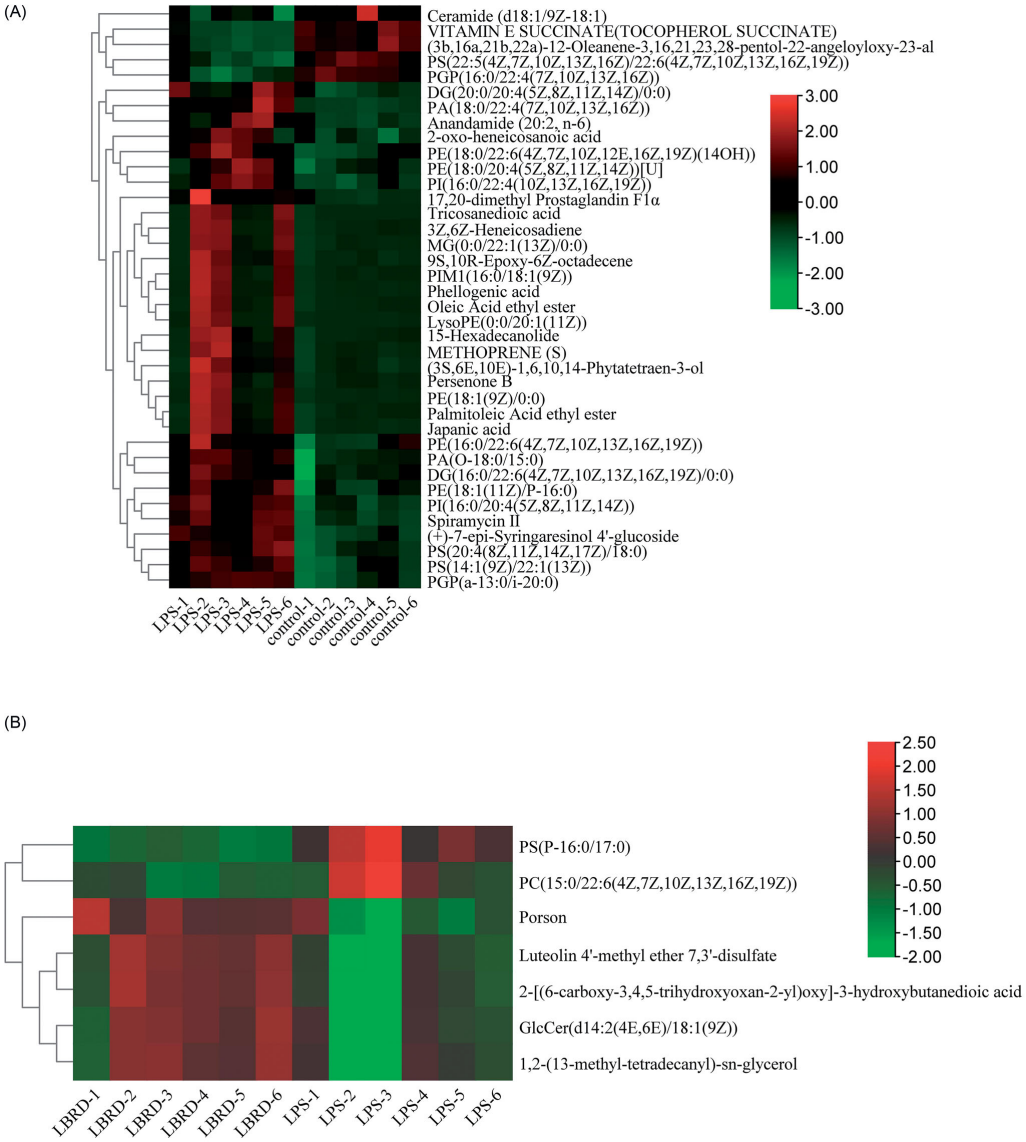

**Figure:**
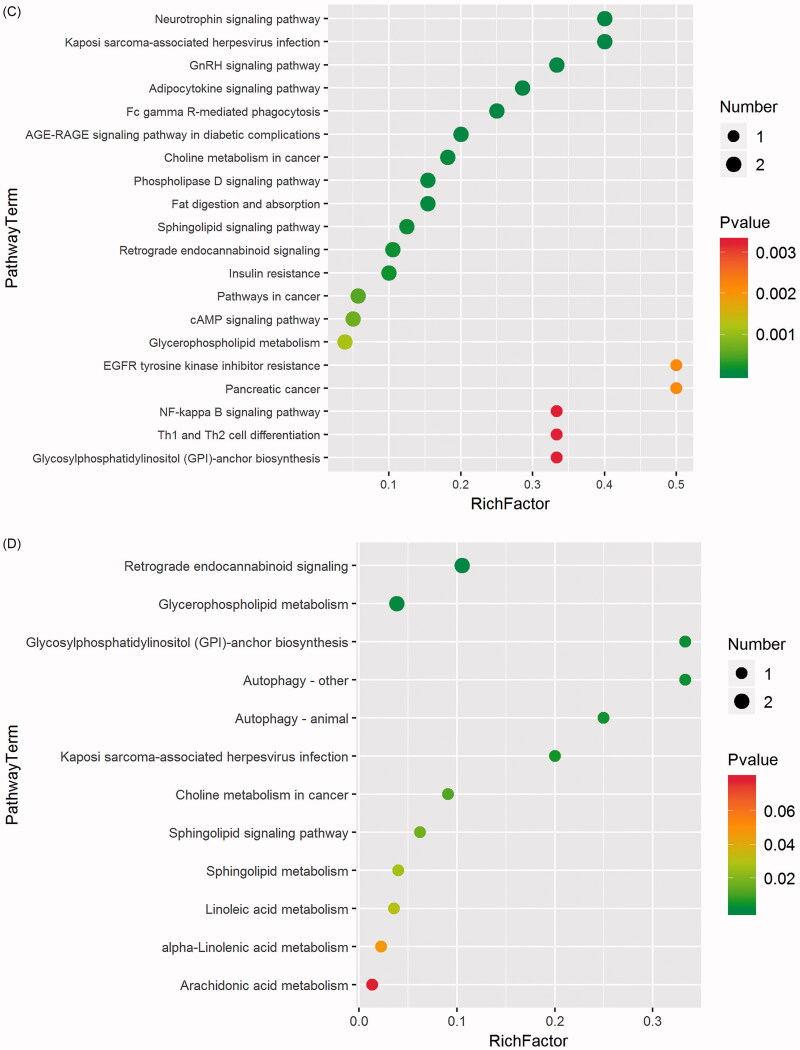

**Table 1.** Comparison of key differential metabolites between the control group and the model group.

Metabolites	Super class	Class	Molecular mass	Average		Fold change (model vs. control)	*p* Value
				Control	Model		
Vitamin E succinate (tocopherol succinate)	Unclassified	Unclassified	553.39	1630.2	5334.04	3.272015704	0.00004
(3b,16a,21b,22a)-12-Oleanene-3,16,21,23,28-pentol-22-angeloyloxy-23-al	Lipids and lipid-like molecules	Prenol lipids	569.38	611.27	1813.6	2.966937687	0.0002
PGP (16:0/22:4(7,10*Z*,13*Z*,16*Z*))	Lipids and lipid-like molecules	Glycerophospholipids	896.54	652.68	1195.2	1.831218974	0.00007
PS(22:5(4*Z*,7*Z*,10*Z*,13*Z*,16*Z*)/22:6(4*Z*,7*Z*,10*Z*,13*Z*,16*Z*,19*Z*))	Lipids and lipid-like molecules	Glycerophospholipids	864.52	262.41	434.24	1.654814984	0.0002
Ceramide (d18:1/9*Z*-18:1)	Lipids and lipid-like molecules	Sphingolipids	586.52	3313.82	5173.05	1.561053407	0.029
DG (16:0/22:6(4*Z*,7*Z*,10*Z*,13*Z*,16*Z*,19*Z*)	Lipids and lipid-like molecules	Glycerolipids	623.5	13224.45	8776.86	0.663684312	0.007
Anandamide (20:2, n-6)	Lipids and lipid-like molecules	Fatty acyls	390.28	6999.05	4536.15	0.648109386	0.008
PE (18:0/20:4(5*Z*,8*Z*,11*Z*,14*Z*)) [U]	Unclassified	Unclassified	790.54	19158.23	11998.59	0.626289067	0.013
PE (18:1(11*Z*)/P-16:0)	Lipids and lipid-like molecules	Glycerophospholipids	746.51	3542.21	2100.78	0.593070428	0.004
PA (O-18:0/15:0)	Lipids and lipid-like molecules	Glycerophospholipids	649.52	2917.46	1664.45	0.570513392	0.001
PS (20:4(8*Z*,11*Z*,14*Z*,17*Z*)/18:0)	Lipids and lipid-like molecules	Glycerophospholipids	810.53	784.44	429.96	0.548110754	0.002
PE (16:0/22:6(4*Z*,7*Z*,10*Z*,13*Z*,16*Z*,19*Z*))	Lipids and lipid-like molecules	Glycerophospholipids	762.51	2317.8	1263.72	0.545223919	0.045
DG (20:0/20:4(5*Z*,8*Z*,11*Z*,14*Z*)/0:0)	Lipids and lipid-like molecules	Glycerolipids	655.56	8049.5	4300.93	0.534310206	0.01
PI (16:0/22:4(10*Z*,13*Z*,16*Z*,19*Z*))	Lipids and lipid-like molecules	Glycerophospholipids	885.55	9801.11	4982.42	0.508352625	0.003
PI (16:0/20:4(5*Z*,8*Z*,11*Z*,14*Z*))	Lipids and lipid-like molecules	Glycerophospholipids	857.52	3209.37	1563.64	0.487210886	0.0004
17,20-Dimethyl prostaglandin F1α	Unclassified	Unclassified	367.28	589.47	284.12	0.481992298	0.04
Spiramycin II	Lipids and lipid-like molecules	Polyketides	883.53	1754.29	812.24	0.463002126	0.00009
(+)-7-*epi*-Syringaresinol 4′-glucoside	Lignans, neolignans and related compounds	Lignan glycosides	598.25	364.56	163.9	0.449583059	−0.00002
15-Hexadecanolide	Phenylpropanoids and polyketides	Macrolides and analogues	253.22	541.42	226.63	0.418584463	0.037
Methoprene (S)	Unclassified	Unclassified	311.26	553.25	217.89	0.393836421	0.03
Palmitoleic acid ethyl ester	Unclassified	Unclassified	281.25	18179.39	6713.7	0.36930282	0.03
PS (14:1(9*Z*)/22:1(13*Z*))	Lipids and lipid-like molecules	Glycerophospholipids	786.53	551.19	203.02	0.36833034	0.0003
PE (18:0/22:6(4*Z*,7*Z*,10*Z*,12E,16*Z*,19*Z*)(14OH))	Lipids and lipid-like molecules	Glycerophospholipids	806.54	808.71	297.25508	0.367566965	0.01141
Japanic acid	Lipids and lipid-like molecules	Fatty acyls	339.29	47407.422	15999.61	0.33749167	0.02
(3S,6*E*,10*E*)-1,6,10,14-Phytatetraen-3-ol	Lipids and lipid-like molecules	Prenol lipids	308.29	499.02	158.26	0.317141598	0.02
Persenone B	Lipids and lipid-like molecules	Fatty acyls	365.3	1816.22	554.05	0.305056656	0.05
PGP (a-13:0/i-20:0)	Lipids and lipid-like molecules	Glycerophospholipids	834.53	199.83	57.72	0.288845519	0.00004
9S,10R-Epoxy-6*Z*-octadecene	Lipids and lipid-like molecules	Fatty acyls	265.25	1011.3705	246.41	0.243639695	0.03069
2-Oxo-tricosanoic acid	Lipids and lipid-like molecules	Fatty acyls	369.34	1167.34	249.02	0.213322597	0.04
MG (0:0/22:1(13*Z*)/0:0)	Lipids and lipid-like molecules	Glycerolipids	395.35	2034.97	419.34	0.20606692	0.03
Tricosanedioic acid	Lipids and lipid-like molecules	Fatty acyls	367.32	16203.6	3135.26	0.19349157	0.03
3*Z*,6*Z*-Heneicosadiene	Lipids and lipid-like molecules	Fatty acyls	337.31	1055.26	202.77	0.192151697	0.04
Oleic acid ethyl ester	Unclassified	Unclassified	309.28	8705.94	1668.2	0.191616299	0.04
PA (18:0/22:4(7*Z*,10*Z*,13*Z*,16*Z*))	Lipids and lipid-like molecules	Glycerophospholipids	797.54	237.85	41.89	0.176119403	0.0005
Phellogenic acid	Lipids and lipid-like molecules	Fatty acyls	353.3	296.31	48.04	0.162127502	0.04
PE (18:1(9*Z*)/0:0)	Unclassified	Unclassified	480.31	9977.1	1523.51	0.152700685	0.04
PIM1 (16:0/18:1(9*Z*))	Lipids and lipid-like molecules	Glycerophospholipids	979.57	2405.35	366.17	0.152231484	0.05
LysoPE (0:0/20:1(11*Z*))	Lipids and lipid-like molecules	Glycerophospholipids	508.34	724.16	76.86	0.106136765	0.04

**Table 2.** Comparison of key differential metabolites between model and the LBRD treatment group.

Metabolites	Super class	Class	Molecular mass	Average		Fold change (LBRD vs. model)	*p* Value
				Model	LBRD		
GlcCer (d14:2(4*E*,6*E*)/18:1(9*Z*))	Lipids and lipid-like molecules	Sphingolipids	650.5	225.19	357.65	1.5882144	0.03
Porson	Phenylpropanoids and polyketides	Diarylheptanoids	387.18	448.84	704.39	1.56935656	0.008
Luteolin 4′-methyl ether 7,3′-disulfate	Lipids and lipid-like molecules	Polyketides	478.01	2224.34	3366.85	1.51364	0.008
1,2-(13-Methyl-tetradecanyl)-*sn*-glycerol	Lipids and lipid-like molecules	Glycerolipids	535.5	213.52	320.34	1.500281	0.04
2-[(6-Carboxy-3,4,5-trihydroxyoxan-2-yl)oxy]-3-hydroxybutanedioic acid	Organic oxygen compounds	Organooxygen compounds	365.01	244.31	353.81	1.44820106	0.01
PS (P-16:0/17:0)	Lipids and lipid-like molecules	Glycerophospholipids	778.56	908.65	573.6	0.63126616	0.0004
PC (15:0/22:6(4*Z*,7*Z*,10*Z*,13*Z*,16*Z*,19*Z*))	Lipids and lipid-like molecules	Glycerophospholipids	790.54	6978.77	4271.7	0.61209927	0.04

**Figure 7.**
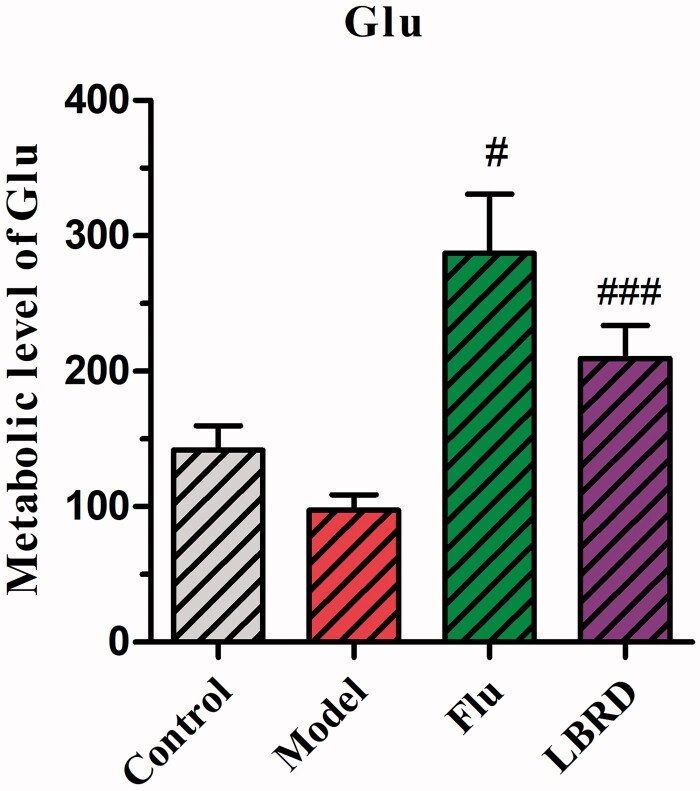
Metabolic level of Glu compared between control group, model group, LPS + LBRD group and LPS + Flu group. The metabolic levels of Glu showing reduction in depressed rats compared with the control group without statistical difference (*p*> 0.05). After the intervention of LBRD, the metabolic levels of Glu in LBRD group, showed significant increasement than model group. *n* = 8–10 per/group. ^#^*p*< 0.05, ^###^*p*< 0.001 vs. the model group.

After analysing the common metabolites associated with depression in the mPFC, we found that the metabolic levels of Glu were decreased in the model group, without significantly statistical difference compared to the control group (Figure 7). After the intervention with LBRD, the metabolic levels of Glu in the LBRD group showed a significant increase compared with the model group (*p* < 0.001, Figure 7), consistent with the change trend of Glu in the serum.

### Metabolic pathway analysis

In this study, we identified several pathways that may be perturbed in LPS-induced depression-like rats. The 20 most significantly different pathways are as follows: (1) neurotrophin signalling pathway; (2) Kaposi sarcoma-associated herpesvirus infection; (3) gonadotropin-releasing hormone (GnRH) signalling pathway; (4) adipocytokine signalling pathway; (5) Fc gamma R-mediated phagocytosis; (6) advanced glycation end product (AGE)-receptor for AGE (RAGE) signalling pathway in diabetic complications; (7) choline metabolism in cancer; (8) phospholipase D signalling pathway; (9) fat digestion and absorption; (10) sphingolipid signalling pathway; (11) retrograde endocannabinoid signalling; (12) insulin resistance; (13) pathways in cancer; (14) cAMP signalling pathway; (15) glycerophospholipid metabolism; (16) epidermal growth factor receptor (EGFR) tyrosine kinase inhibitor resistance; (17) pancreatic cancer; (18) NF-κB signalling pathway; (19) T-helper type 1 (Th1) and T-helper type 2 (Th2) cell differentiation cell differentiation; and (20) glycosylphosphatidylinositol (GPI)-anchor biosynthesis. The detailed results of pathway analysis are shown in Figure 6(C).

To further explore the underlying mechanism of the perturbed metabolites induced by the LPS model and treatment with LBRD, a comprehensive metabolic network was mapped using the KEGG database. By doing this, 12 nominally significantly altered canonical pathways were identified (Figure 6(D)). The most significantly altered canonical pathways were (1) retrograde endocannabinoid signalling; (2) glycerophospholipid metabolism; (3) GPI-anchor biosynthesis autophagy; (4) autophagy – other; (5) autophagy–animal; (6) Kaposi sarcoma-associated herpesvirus infection; (7) choline metabolism in cancer; (8) sphingolipid signalling pathway; (9) sphingolipid metabolism; (10) linoleic acid metabolism; (11) α-linolenic acid metabolism; and (12) arachidonic acid metabolism.

After comparing and analysing the metabolic network between the control rats, LPS-induced depression-like rats and LBRD-treated rats, we determined the five most significant pathways associated with the occurrence and the treatment of depression: retrograde endocannabinoid signalling, glycerophospholipid metabolism, GPI-anchor biosynthesis, autophagy and Kaposi sarcoma-associated herpesvirus infection, which are probably associated with the biological functions of the nervous system signalling/neurotransmitters, lipid metabolism, autophagy and virus infection, suggesting that LBRD may play an antidepressant role through these pathways.

## Discussion

As a common mental disorder, depression is a multifactorial disease with complex pathological mechanisms that involve complex interactions between genes and the environment, often accompanied by many abnormal changes in molecules and signalling pathways (Lao et al. 2020). Undoubtedly, depression has become a public health issue attracting increasing attention.

LPS, a highly effective and powerful inducer of inflammatory cytokines (such as TNF-α, IL-1 and IL-6), is often used to construct a model of depression by activating the peripheral or central innate immune systems (O'Connor et al. 2009; Wu et al. 2016). The LPS-induced animal model, which is adopted to clarify the relationship between the immune system and depression symptoms, has been repeatedly proven to be an effective and predictive animal model of depression (Henry et al. 2008). Although, multiple studies have focussed on inflammation-related neuro-behavioural changes in LPS-induced depression-like rats, only a few studies have concentrated on the method of metabolism to assess differential metabolites changes in the mPFC of LPS-induced depression-like rats.

SPT, FST and EPM are common behavioural tests for depression. In this study, behavioural tests demonstrated that LBRD and fluoxetine have similar efficacy in changing depressive behaviours. Thus, LBRD may be an effective antidepressant with a strong efficacy and high safety.

Multiple studies have demonstrated that the complex mechanism of depression is mainly related to monoamine neurotransmitters, inflammation and oxidative stress response, HPA axis dysfunction, synaptic plasticity, neurotrophic factor secretion disorder, circadian rhythm disorder and so on (Lindqvist et al. 2017; Erjavec et al. 2021). Studies have shown that the abnormalities of various serotonergic activity in serum, such as deranged 5-HT synthesis, release, reuptake and metabolism may initiate or trigger depression (Dell’osso et al. 2016). Previous research has confirmed that the altered levels of the major excitatory and inhibitory systems in the brain are the main changes in patients with major depressive disorder (Duman et al. 2019). Glu and GABA are neurotransmitters that are abundant in the central nervous system (CNS), particularly in the cerebral cortex. GABA is produced by Glu in the brain cells and plays a role in the inhibition of neurotransmitters. After the intervention of LBRD, it was found that the levels of the primary excitatory neurotransmitter Glu and inhibitory neurotransmitter GABA were reversed. Research shows that neuroinflammation, which affects synaptic function, is considered to be the pathogenesis and potential therapeutic target of many neuronal diseases, including depression (Habbas et al. 2015; Kaufmann and Menard 2018). It has been shown that LPS not only causes depressive behaviours but also activates microglia and induces neuroinflammation in the CNS (Murray et al. 2011; Pascual et al. 2012). Meanwhile, the increase of body temperature in rats is often associated with LPS injection. Thyroid hormone is an important hormone that promotes metabolism and regulates basal body temperature. Based on our previous studies, we concluded that the elevated levels of T4 and TRH could be used as indicators of deficiency heat syndrome, and the LPS-induced depression model may have symptoms similar to those of lily disease. Furthermore, CORT, a biorhythm marker, has been shown to increase after the occurrence of depression. In line with the above conclusions, our behavioural tests and ELISA results demonstrated that LPS could successfully induce a depression model with the symptoms similar to those of lily disease, and LBRD could reverse depression based on its complex aetiology of depression.

According to the change of Glu in metabolites and serum, the intervention of LBRD could reverse the decreased Glu level induced by LPS. Without doubt, there is always a controversy that the expression level of Glu in depressed patients and depression animal models might be increased or decreased. The reasons for these discrepancies are unclear, but might be related to the length of stress exposure, type of stressors, causes of depression, and differences in samples such as blood, cerebrospinal fluid and different brain tissue. The test results might be variant, even in the same brain area. Thus, we could conclude that the mechanism of depression is very complex, and it is difficult to find a cure for this disease without fully deciphering the underlying mechanism. Therefore, it is crucial to identify the key molecules with potential antidepressant activity.

Metabolomics is a newly developed quantitative analysis of small molecules (<1500 Da) in biological systems to explore and establish the direct relationship between the change in metabolic content and the change in biological phenotype through the overall analysis of endogenous low-molecular-weight metabolite content, in order to search for the relationship between metabolites and physiological or pathological changes (Dettmer and Hammock 2004; Dunn et al. 2011; Oscar et al. 2019). At the same time, the method of metabolism is consistent with the characteristics of multiple components, multiple targets and overall regulation in TCM, and can comprehensively evaluate therapeutic effects and quantify metabolic variability, making it suitable for evaluating the overall synergistic effects of TCM (Pferschy-Wenzig et al. 2017; Zhang et al. 2018; Gong et al. 2019). In recent years, an increasing number of studies on depression have applied metabolomics to analyse the pathogenesis, the metabolic pathways and the biomarkers for depression diagnosis. Previous research has focussed on the rat model of chronic stress depression (Liu et al. 2020). This study differs in that we concentrate on the metabolites in rats with LPS-induced depression.

In our study, five pathways which are most significantly associated with the occurrence and treatment of depression, including retrograde endocannabinoid signalling, glycerophospholipid metabolism, GPI-anchor biosynthesis, autophagy and Kaposi sarcoma-associated herpesvirus infection are related to the biological functions of the nervous system signalling/neurotransmitters, lipid metabolism, autophagy and virus infection.

Research has demonstrated that the changes in depolarization-induced suppression of excitation or depolarization-induced suppression of inhibition, and the occurrence of long-term depression could be induced by retrograde endocannabinoid signalling (Adermark and Lovinger 2007). The expression of the endogenous cannabinoid system in the limbic regions of the brain regulates negative emotions and stress responses, while a decrease in endogenous cannabinoid system function may be a predisposing factor for severe depression, wherein insufficient endogenous cannabinoid signalling may lead to depressive and anxious behaviour (Yu et al. 2012). Previous research has also demonstrated that the imbalance of retrograde endocannabinoid signalling is related to a variety of CNS and immune system diseases (Lowe et al. 2021), which is consistent with our findings.

Simultaneously, glycerophospholipid metabolism has been shown to be closely related to cognitive impairment induced by depression. Studies have shown that abnormalities in lipid metabolism, especially fatty acid and glycerophospholipid metabolism, caused by increased cholesterol and decreased reabsorption, are involved in the pathophysiological process of depression (Zhang et al. 2018; Ye et al. 2020). Glycerophospholipids are associated with neuronal function, and affect perception and emotional behaviour, resulting in depression and anxiety disorders. Depression is characterized by disturbances in the central lipid metabolism.

Autophagy is considered to be associated with stress-related diseases, such as depression. Several studies have demonstrated the effects of antidepressant drugs or compounds on autophagy (Gassen and Rein 2019). However, the role of autophagy in depression remains controversial (Zhang et al. 2020). On the one hand, some antidepressant drugs could relieve the depression manifestations by enhancing hippocampal autophagy through the brain-derived neurotrophic factor (BDNF)–tyrosine kinase receptor B (TrkB) pathway (Liu et al. 2020). On the other hand, the antidepressant effect via upregulation of the BDNF–TrkB pathway attenuated autophagy in the hippocampus (Song et al. 2017). Therefore, the therapeutic effect of LBRD via the above metabolic pathways is probably associated with the biological functions of the nervous system signalling/neurotransmitters, lipid metabolism, autophagy and virus infection, in accordance with some results on neurotransmitter levels.

Combined with changes in metabolites and metabolic pathway analysis, we proposed that glycerophospholipid metabolism is involved mostly in the occurrence and treatment of depression. Based on the levels of neurotransmitters, metabolites and network pharmacology analysis, glutamatergic synapses may be one of the major targets, and LBRD works on them to exert a combined antidepressant effect in LPS-induced depression-like rats.

## Conclusions

**Figure 8.**
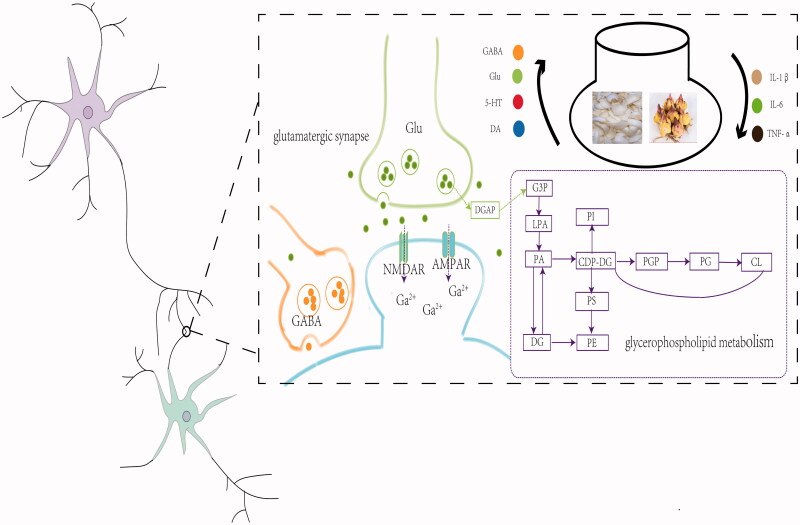
The mechanism underlying the treatment of LBRD on LPS-induced depression-like rats. Glycerophospholipid metabolism, and glutamatergic synapse may be the potential pathway for LBRD to exert the antidepressant effect on the LPS-induced depression-like rats. At the same time, LBRD increases the levels of GABA, Glu, 5-HT and DA, and decreases the levels of IL-1β, IL-6 and TNF-α.

Collectively, the characteristics of multiple compounds and targets of LBRD have been demonstrated. According to our study, the occurrence and improvement of depression are associated with the neurotransmitter and proinflammatory cytokine levels. Glycerophospholipid metabolism, and glutamatergic synapse may be the potential pathways for LBRD to exert an antidepressant effect on LPS-induced depression-like rats (Figure 8). However, the metabolism biomarkers, and the mechanism underlying the treatment of LBRD still need to be further studied using metabolomic technology to guide the clinical application and modernization of TCM classical formulas.

## Data Availability

The raw data supporting the conclusions of this manuscript will be available from the corresponding author on reasonable request.
